# Pharmacological History of Missing Subjects: Perspective of a Correction Factor to Aid in the Study of Bone Remains

**DOI:** 10.3390/biology11081128

**Published:** 2022-07-27

**Authors:** Camilla Cecannecchia, Benedetta Baldari, Andrea Cioffi

**Affiliations:** Department of Anatomical, Histological, Forensic and Orthopaedic Sciences, Sapienza University of Rome, 00161 Rome, Italy; camilla.cecannecchia@uniroma1.it (C.C.); benedetta.baldari@uniroma1.it (B.B.)

**Keywords:** forensic anthropology, drugs, identification, forensic science

## Abstract

**Simple Summary:**

The reconstruction of the biological profile of skeletal remains of missing subjects is also based on the analysis of the quality of bone tissue. The density of bone mass is a factor that allows us to inscribe the subject in a specific age group. Bone density varies not only according to age but also by the intake of certain drugs and certain abuse substances. The objective of our study is to propose the introduction of pharmacological history in the profile of missing persons. Information on drugs or abuse substances taken by the missing person is a useful corrective factor for tracing the chronological age of bone remains found, increasing the likelihood of identification. We emphasize the usefulness of this information also for the characterization of bone injuries and for the dating of antemortem fractures, useful elements to trace the cause and dynamics of death. The evaluation of these findings is also based on the characteristics of the bone tissue of skeletal remains which is also affected by any drugs and/or substances of abuse. Therefore, we believe that the pharmacological history of the missing subjects could be a new and interesting tool to help the activity of the forensic anthropologist.

**Abstract:**

In forensic anthropology, bone mineral density and the estimation of the dating of fractures based on the degree of progress of healing processes are important parameters of study on bone remains. With our article we aim, on the one hand, to highlight the importance that these parameters have in the reconstruction of the biological profile of the subject, as well as the time and the cause of death; on the other hand, we aim to limit their variability according to the medical substances and/or abuse assumed during life by the subject. The aim of this article is to encourage the introduction of the pharmacological history of missing persons as a new correction factor for the study of bone remains, possibly based on new scientific studies that allow us to establish with greater specificity the effect that certain pharmacological therapies produce on bone mass and the speed of remodeling.

Forensic anthropology is a highly dynamic and evolving branch of forensic science that deals with the study of bone remains in the context of judicial investigations, in order to trace the identity of the deceased as well as the cause, means and dynamics in which the death occurred. In fact, forensic anthropologists, even if not involved in the cause of death, are often entrusted with the onerous responsibility of reconstructing the events that preceded the death. Consider the key role played by these experts in mass disasters [1,2], in crime scenes involving charred corpses [3], and in investigations of crimes against humanity [4].

The boundaries of competence of forensic anthropology have expanded radically since the early pioneers of this discipline began to apply science, anatomy, and physical anthropology to the medical forensic field. In fact, if originally the area of expertise of this branch was mainly focused on the identification of human remains, in recent years it has specialized in the analysis of traumas—especially of skeletal interest—found on remains.

Forensic anthropologists have become ever more skilled in biomechanics and the dynamics of traumatic bone injury, and in the bone remodeling system and its variables; this makes them experts in classifying these lesions qualitatively and to place them chronologically with respect to death (ante-mortem, perimortem, post-mortem) [5].

Moreover, in recent years, forensic anthropology is refining its field of expertise through the application of new technologies and interdisciplinary collaboration with specialized engineers [6,7]. The new technologies allow us to build—with increasing scientific reliability—the biological profile of the subject (traditionally based on the estimation of the age at the time of death, on the determination of sex and of ethnicity, etc.) by also identifying the thickness of facial soft tissues in facial reconstruction [8]. However, it should be pointed out that the morphological and osteometric data useful in the reconstruction of the biological age of the subject (for example, the degree of maturation of the bones, the modifications of the articular surfaces, the welding of the cranial sutures, the dental development) suffer from many variables related not only to chronologic age and genetic factors, but also to living habits, work activities, eating habits, drugs and any abuse substances taken in life [9]. These variables can also decisively affect the placement of bone remains in one age class rather than another, compromising their identification. In addition, these factors—acting on the skeletal tissue and its metabolism—can contaminate the results of the analyses performed on bone traumas by the forensic anthropologist, which are of key importance for the subsequent definition of the cause of death. In fact, each age class is associated with both a different degree of bone density, of toughness cortical bone and a different rate of bone remodeling, variables that in life affect the resistance to trauma and the bone healing [10,11,12,13,14,15].

Therefore, inserting unknown human remains (due to bone characteristics affected by other factors, including drugs taken during life) in an incorrect age class can also adversely affect the interpretation of fractures that may be found on them. For example, the ‘age factor’ affects the quality of human bone tissue and it is used, in forensic anthropology, as a correction factor for the interpretation of injuries attributed to blunt force trauma (BFT). The characteristics of these injuries depend not only on the type of weapon used and the impact mechanism, but also on factors related to the victim, including age [16]. Different age groups are associated with different trauma resistance and bone density [17].

Therefore, by not considering other factors that alter the bone mineral density, there is the risk of inserting the unidentified subjects in a wrong age group, compromising the identification and characterization of any skeletal trauma suffered during life.

In addition, the degree of bone remodeling—age correlated and directly proportional to the rate of repair of the fracture—may be affected by the lifetime intake of drugs or abuse substances [18]. This parameter is also fundamental for the forensic anthropologist because it is useful to estimate the possible survival time of the person elapsed between the skeletal trauma and the death occurred. Traumatic skeletal injuries occurring in the antemortem period are characterized by the presence of remodeling or healing of the fracture. In children, even injuries that occurred a few days before death may still show signs of periosteal reactivity, while in adults it is between 10 and 14 days [19]. In adults, within the next 40–90 days, the bone callus is formed, while the final remodeling and healing of the fracture is completed within 1–2 years. The time of bone remodeling, and in particular of bone callus formation, is highly variable and is influenced by the subject’s health conditions, environmental factors and genetic factors [20]. It is crucial to study all possible intrinsic variables of bone remodeling time since fracture dating is important to reconstruct the survival time of a subject between trauma and death. This is essential, for example, in allowing forensic anthropologists to shed light on possible torture dynamics.

They are in fact characterized by offensive injuries repeated over time and aimed at the mere psycho-physical suffering of the victim. Such elements are indispensable in war contexts when the forensic anthropologist—as has happened in the past [21]—oversees investigating evidence of international crimes on the bone remains of victims.

Therefore, knowing the variables that influenced the bone density and the rate of bone remodeling of the subject ante-mortem, using them to correct the results of the investigations of the bone remains could be a useful support to the activity of the forensic anthropologist.

Drugs are among the extrinsic factors that in life most affect bone density and the degree of bone remodeling; moreover, in contrast to other variables that also affect them—such as physical activity and nutritional habits—the action of drugs on bone tissue could be qualitatively and quantitatively estimated with greater specificity. In particular, the effect that a given drug therapy produced during life on the skeleton could be obtained not only on the basis of the type and number of drugs taken, but also on the basis of the duration of administration and dosage. Indeed, several drugs are associated with a reduction in bone mineral density and a risk of early osteoporosis [9]. These include, for example, antipsychotic drugs that can cause hyperprolactinemia [22], a condition associated with a reduction in bone mass density (BMD) [23]. Drugs against diabetes are associated with a risk of pathological fractures (and, therefore, osteoporosis) variable depending on the type of drug used, but regardless of the duration of exposure to the same [24]. Chronic use of anti-epileptic drugs (AED) also appears to be associated with skeletal abnormalities, mainly due to impaired bone turnover and reduced bone mass density [25]. Anticoagulants (Heparin and Vitamin K Antagonists), GnRH Agonists (Gonadotropin Releasing Hormone Agonists), cytotoxic drugs (e.g. methotrexate), and glucocorticoid therapies lead to a reduction in bone mass. Glucocorticoids, even if administered by os and at low doses (5 mg/day) involve a loss of BMD at 1 year, while this would tend to increase again after about 1 year from cessation when the risk of fracture is reduced [26,27,28]. Additionally, the intake of abuse substances, such as amphetamines, can cause a reduction in bone density [9]. Cocaine—on the basis of the route of administration—can result in lithic bone lesions (such as perforation of the nasal septum, lesions of the palate with oro-nasal fistulas, lesions of the maxillary sinus, etc.) which, not usually accompanied by repair with newly formed bone tissue, may direct the forensic anthropologist to the suspicion of bone remains of a person who is a cocaine user [9,29].

These and other medicinal or abuse substances have a variable effect on bone tissue in terms of quality and quantity, but may significantly affect the results of bone remains studies.

Therefore, skeletal indicators (both those useful for tracing the biological identity of the subject and those useful for reconstructing the dynamics and means of death) should, where possible, be corrected on the basis of the pharmacological history of the subject. This, in concert with all the other extrinsic variables (habits of life, pathologies suffered in life, nutrition), would allow us to narrow the field in the comparison between bone remains and missing persons and to reconstruct, with a greater level of specificity, the dynamics and the cause of death. It is, therefore, desirable to investigate the qualitative and quantitative effect that different drugs produce on bone tissue in life, in relation to the importance that this variable could play in the forensic anthropology field. Knowing the effects that various drugs have, globally, on the bones of subjects—in relation to specific pharmacodynamic and pharmacokinetic variables—will positively affect the evolution of forensic anthropology and its applications. Once in-depth and detailed knowledge has been acquired, it will be essential to know—when possible—the pharmacological history of the missing subjects in order to verify the specific correspondence with the results of bone remains analysis. Finally, in order to achieve this aim, it would be necessary that the organizations dealing with missing persons should include the pharmacological history among other relevant physical and biological information.

## Data Availability

Not applicable.
